# A One Pot Method for Preparing an Antibacterial Superabsorbent Hydrogel with a Semi-IPN Structure Based on Tara Gum and Polyquaternium-7

**DOI:** 10.3390/polym10070696

**Published:** 2018-06-22

**Authors:** Jie Shen, Bingjie Li, Xianxu Zhan, Lijuan Wang

**Affiliations:** 1 Key Laboratory of Bio-based Materials Science and Technology of Ministry of Education, Northeast Forestry University, Harbin 150040, China; sj@nefu.edu.cn (J.S.); binaca@126.com (B.L.); 2 Research Center of Wood Bionic Intelligent Science, Northeast Forestry University, Harbin 150040, China; 3 Dehua TB New Decoration Material Co., Ltd., Huzhou 313218, China; zhanxianxu@126.com

**Keywords:** superabsorbent hydrogel, tara gum, polyquaternium-7, antibacterial property

## Abstract

An antibacterial superabsorbent polymer was prepared by graft polymerization of acrylic acid onto tara gum polysaccharide, by adding *N*,*N*-dimethyl-*N*-2-propenyl-2-propen-1-aminium chloride and a polymer with 2-propenamide (polyquaternium-7, PQ7) as an antibacterial agent. The effects of the amount of PQ7 in the hydrogel on its swelling ratio were investigated and maximum swelling ratios of 712 g/g and 68 g/g, in distilled water and 0.9 wt % NaCl solution were attained with 0.5 g PQ7 per gram of tara gum. The superabsorbent hydrogel was characterized by using Fourier transform infrared spectroscopy, X-ray diffraction, scanning electron microscopy and thermal gravimetric analysis. The results showed that poly (acrylic acid) was successfully grafted onto tara gum and a three-dimensional network structure formed with PQ7 chains penetrated in the networks. The antibacterial properties of these superabsorbent hydrogels against *Staphylococcus aureus* and *Escherichia coli* improved with increasing PQ7 content. This study demonstrates a method of preparing novel functional superabsorbent hydrogels.

## 1. Introduction

Superabsorbent polymers (SAP) are three-dimensional networks of hydrophilic polymers connected by chemical and physical crosslinking that can absorb and retain large volumes of water or saline solution as high as 10–1000 times their own weight [1,2]. Typical superabsorbent hydrogels are generally prepared by polymerization or copolymerization of hydrophilic monomers such as acrylic acid, acrylamide, acrylonitrile, methacrylic acid and sodium acrylate which have hydrophilic functional groups like amines, carboxylic acids, hydroxyl groups, amides and sulfonic acid groups [3]. Because of good water absorption, they are widely used in biomedical applications, biosensors, agriculture, catalyst supports, and sorbents for the removal of heavy metal ions [4,5,6,7,8,9]. Although these hydrogels, based on vinyl monomers, have good water absorption, their application is limited because they are not biodegradable and they lack antibacterial activity. To solve the environmental problem which is caused by the non-degradable superabsorbent hydrogels, many researchers have sought to address this environmental problem by preparing superabsorbent hydrogels based on natural materials such as xanthan gum, guar gum, carrageenan and cellulose, and have reported their partial degradation [4,10,11,12,13,14,15]. These studies show that graft copolymerization of vinyl monomers onto polysaccharides is an efficient method for the preparation of superabsorbent hydrogels based on natural materials. Yoshimura et al. used cotton fiber and succinic anhydride as raw materials to prepare a superabsorbent resin with high water absorption and demonstrating substantial degradation under natural conditions in 25 days [16].

Although health care and medical technology have made significant progress, many pathogenic microorganisms remain a major public health threat [17]. Naturally-based superabsorbent hydrogels have attracted much attention in medical and pharmaceutical fields because they are non-toxic, biocompatible and biodegradable [18]. The applicability of these hydrogels could be significantly improved if they also possessed antibacterial properties. There have been many studies aimed at the production of antibacterial naturally-based superabsorbent hydrogels [19,20,21,22]. As yet, most of these studies have involved adding metal nanoparticles to hydrogels as antibacterial agents. Mohan et al. prepared antibacterial hydrogels containing silver nanoparticles [23]. Mehdi et al. synthesized an antibacterial hydrogel containing CuO nanoparticles [24]. However, nanoparticles are prone to agglomeration which leads to uneven dispersion in hydrogels and this phenomenon can greatly affect their antibacterial properties. Metal nanoparticles are too small to be stably presented in the network structure. In addition, these metal nanoparticles are inorganic and cannot be degraded in the environment. Some antibacterial hydrogels contain antibiotics whose antibacterial activity is high [25,26]. However, antibiotics are easy to escape from the hydrogels and can develop drug resistance for the human body. Polyquaternium-7 is a polymeric antibacterial agent which is bio-degradable, and can produce stable mixtures with SAPs. It was therefore chosen to use in the superabsorbent hydrogels in this study.

Polyquaterniums are polycationic polymers and some have antimicrobial properties. The various polyquaterniums are distinguished by a numerical value, which is not related to their chemical structure but to the order in which they were registered [27]. Polyquaternium is a kind of cationic polymer whose cations can attract negatively charged bacteria through the electrostatic force, hydrogen bonds and other forces to inhibit the growth of bacteria [28,29]. Polyquaternium-7 (PQ7) is a kind of polymeric quaternary ammonium salt, derived from acrylamide and dimethyl diallyl ammonium chloride monomers [30]. PQ7 is non-toxic and can be easily dissolved in water which means it can be readily used in the preparation of antibacterial hydrogels. It has a long chain structure which means it can be stably integrated within the network structure of superabsorbent hydrogels. Tara gum (TG) is a low cost polysaccharide extracted from the seeds of the Tara tree. The principal component consists of a linear β-(1–4) mannan backbone with α-d-galactose branches attached by (1–6) linkages, with a ratio of mannose to galactose of 3:1 [31]. Tara gum is mainly used as food additive, such as thickeners, gelling agents and stabilizers for dairy products and frozen products while high-yielding tara gum is only used in this way [32]. Therefore, there is a need to find new potential applications for Tara gum. Abd Alla et al. synthesized SAPs based on TG and poly (acrylic acid) (PAA) by gamma radiation [31]. In the present study, we used the simpler method of aqueous solution polymerization to prepare an antibacterial hydrogel based on TG and PAA with PQ7 incorporated in the network.

In this study, the TG-g-PAA/PQ7 superabsorbent hydrogels were prepared from TG grafted with acrylic acid, with PQ7 blended into it. We investigated the effect of the mass ratio between TG and PQ7 in the hydrogel on its swelling ratio in distilled water, and 0.9 wt % NaCl solution. The hydrogels were characterized using Fourier transform infrared spectroscopy (FTIR), X-ray diffraction (XRD), scanning electron microscopy (SEM) and thermal gravimetric analysis (TGA). We tested the antibacterial activity of the hydrogels against *Staphylococcus aureus* and *Escherichia coli*. This work is significant to the study of superabsorbent resins and to expanding the sources of cosmetics and public health.

## 2. Materials and Methods

### 2.1. Materials

Tara gum (TG) was obtained from Dymatic Fine Chemical Co., Ltd. (Guangzhou, China). Acrylic acid (AA) was purchased from Tianli Chemical Reagent Co., Ltd. (Tianjin, China), and was purified by activated carbon before use. Sodium hydroxide (NaOH) was purchased from Guangfu Technology Development Co., Ltd. (Tianjin, China). Potassium persulfate (KPS) was provided by Sitong Chemical Plant (Tianjin, China). N, N-methylene bisacrylamide (NMBA) was provided by Regent Chemicals Co., Ltd. (Tianjin, China). Polyquaternium-7 (PQ7, 10 wt % in water) was provided by Hangzhou Yinhu Chemical Co., Ltd. (Zhejiang, China). *Staphylococcus aureus* (*S. aureus*) and *Escherichia coli* (*E. coli*) were purchased from Haibo Co., Ltd. (Shandong, China).

### 2.2. Synthesis of TG-g-PAA/PQ7 Superabsorbent Polymers

A solution of purified acrylic acid (AA, 100%), NaOH (2 mol/L), *N*,*N*′-methylenebis-acrylamide (NMBA) (1 g/100 mL) as a crosslinking agent was prepared by agitation in an ice bath. After sufficient stirring, PQ7 (10 wt % in water) was added and the mixture was placed in an ice bath. TG (1 g) and 20 mL distilled water were added into a four-neck round bottom flask and stirred under a nitrogen atmosphere in the water bath at 60 °C. Potassium peroxodisulfate (KPS) as initiator (2 g/100 mL) was added into the flask after 20 min stirring. Then, after a further 15 min, the above cold solution was added. The product was obtained after about 75 min and was then dried at 60 °C after soaking in ethanol. The dried product was crushed to a uniform particle size for characterization. The amount of PQ7 (10 wt % in water) added to the hydrogels was 0, 0.3, 0.4, 0.5, 0.6, and 0.7 g and the products were designated as SAP0, SAP3, SAP4, SAP5, SAP6, and SAP7, respectively.

### 2.3. Characterization

#### 2.3.1. Swelling Behavior

A 200-mesh nylon sieve pouch with 80 to 120 mesh superabsorbent hydrogel powder was immersed into a certain quality of distilled water. The pouch with powder was taken out from the water after a period of time and then stood for 30 min to filter out the excess water through natural filtration. After that, the swollen hydrogel was weighed. The swelling ratio was calculated by the equation as follows:

Q = (m_1_ − m_2_ − m_3_)/m_3_ (1)

where Q is swelling ratio (g/g), m_1_ is the weight of the swollen hydrogel (g), m_2_ is the weight of the nylon sieve pouch (g), and m_3_ is the weight of the dry hydrogel (g). The method of swelling in 0.9 wt % NaCl solution test was the same as swelling in distilled water.

#### 2.3.2. FTIR Spectroscopy

FTIR spectra were conducted on a Nicolette 6700 spectrometer (Thermo Fisher Scientific Co., Ltd., Waltham, MA, USA) with attenuated total reflection (ATR) mode in the range of 4000–600 cm^−1^ at a resolution of 4 cm^−1^.

#### 2.3.3. X-ray Diffraction

XRD patterns of hydrogels were analyzed using a D/max-2200 diffractometer (Rigaku, Osaka, Japan) at a voltage of 40 kV and a current of 30 mA using Cu-Kα radiation. The scanning scope of 2θ ranged from 5 to 40° at a scanning rate of 5°/min.

#### 2.3.4. Scanning Electronic Microscopy

Morphology of the samples were examined under a Quanta 200 scanning electron microscope (Philips-FEI Co., Amsterdam, The Netherlands) with an accelerating voltage of 5 kV. The swollen hydrogel were freeze-dried and then coated with a thin gold layer prior to observation.

#### 2.3.5. Thermogravimetric Analysis

TGA of the samples was carried out on a TA Instruments TGA Q500 thermal gravimetric analyzer (TA Instruments, New Castle, DE, USA). The heating rate was 10 °C/min in the temperature range from 20 to 600 °C under nitrogen atmosphere.

#### 2.3.6. Antibacterial Activity

Antibacterial effects of the samples against both *S. aureus* (gram-positive) and *E. coli* (gram-negative) were determined according to agar diffusion test. Samples were pressed into tablets of 1 cm and placed on the agar plate to expose to bacteria in solid media. The agar plates with samples were incubated at 37 °C for 24 h. The inhibition zone around each sample was measured and recorded as the antibacterial effect of SAPs.

#### 2.3.7. Mechanical Property Test

The hydrogel samples were cut in sizes of 25 mm × 25 mm × 10 mm for modules tests. The test was conducted by using an A1-7000S universal material testing machine (Gotech Testing Machines INC, Dongguan, China) at a rate of 1.00 mm/min.

## 3. Results and Discussion

### 3.1. Mechanism of the Superabsorbent Polymer

**Figure 1:**
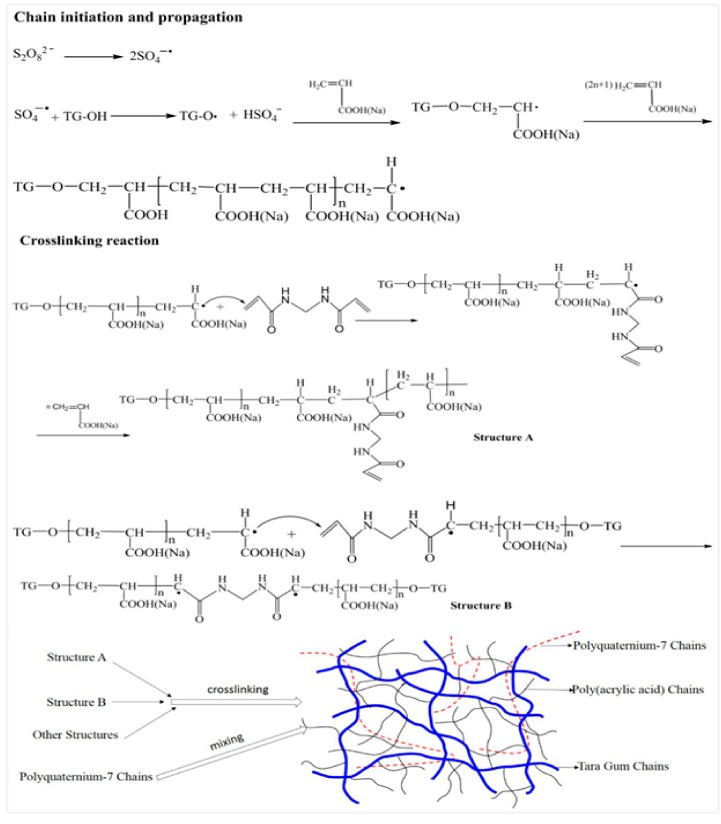
Synthesis mechanism of the antibacterial SAP.

The superabsorbent hydrogel network of TG-g-PAA/PQ7 is formed by graft copolymerization of AA monomers onto TG macromolecular chains, followed by the addition of PQ7, in the presence of KPS and NMBA. The initiator KPS is decomposed under heating to produce a high concentration of sulfate anion radicals, and these radicals strip hydrogen atoms from the -OH groups of TG to form TG radicals. These TG radicals act as active sites during the graft copolymerization and initiate acrylic acid monomers into chain propagation. During the chain propagation, the crosslinking agent NMBA, with its dual vinyl functions, takes part in the polymerization reaction so that a superabsorbent hydrogel is formed with a crosslinked network structure [33]. NaOH was added to neutralize the -COOH to form -COONa which increased the osmotic pressure during the water-absorbing process. The degree of neutralization has a significant effect on the swelling ratio [34]. Since PQ7 chains do not have reactive groups which can react with AA or TG, they do not undergo chemical reaction except for the physical entanglement during the formation of the polymer. The network structure is formed between PAA and TG, while PQ7 chains are merely entangled in the three-dimensional network. The network and PQ7 maintain their own relatively independent chemical stabilities. FTIR results show that there is no new chemical bonds formed between PQ7 chains and TG or PAA. Thus, acrylic acid is polymerized and crosslinked in the presence of TG chains and PQ chains entangle in the network which means that a semi-interpenetrating network structure (semi-IPN) has been formed [31]. The formation of the structure corresponds to the results of XRD analysis and the SEM pictures. Figure 1 shows the reaction process and the schematic diagram of the network.

### 3.2. Swelling Behavior

**Figure 2:**
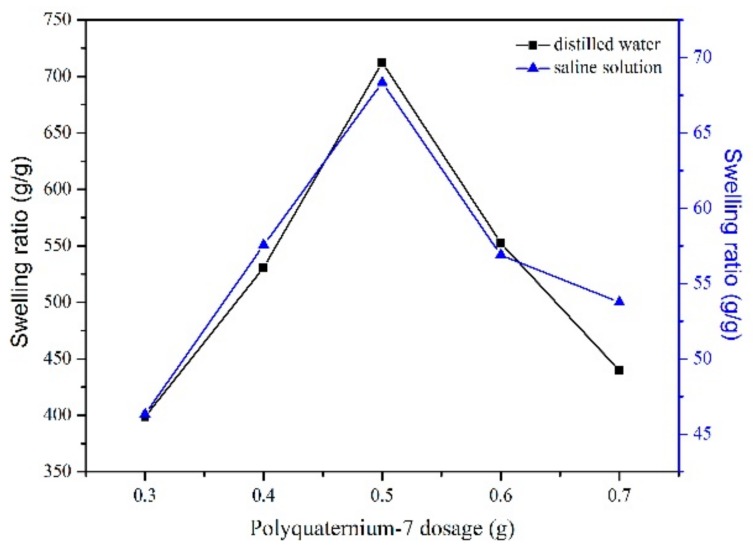
Effect of PQ7 dosage on the absorbency.

The swelling behavior of the superabsorbent hydrogel is shown in Figure 2. It can be seen that the swelling ratios of TG-g-PAA in water and 0.9 wt % NaCl are both high. Thus TG-g-PAA superabsorbent hydrogel has a good water absorption without the addition of PQ7. The addition of increasing amounts of PQ7 results in the swelling ratio decreasing first and then increasing to a maximum. After the maximum, the swelling ratio decreases again. This behavior is attributed to the long chain structure of PQ7. When the amount of PQ7 is small, the intermolecular forces are weak and the long chain can move freely, this affects the polymerization reaction and causes the network structure to be incomplete. With increased PQ7 dosage, the intermolecular force increases and the long chain can no longer move freely. The negative impact of PQ7 on the formation of network structure decreases so that the swelling ratio increases. However, when PQ7 is in excess, the concentration of the polymer becomes high such that the space in the polymer is limited and the ductility of the long PQ7 chain is reduced which causes a decrease in swelling ratio.

### 3.3. FTIR Analysis

**Figure 3:**
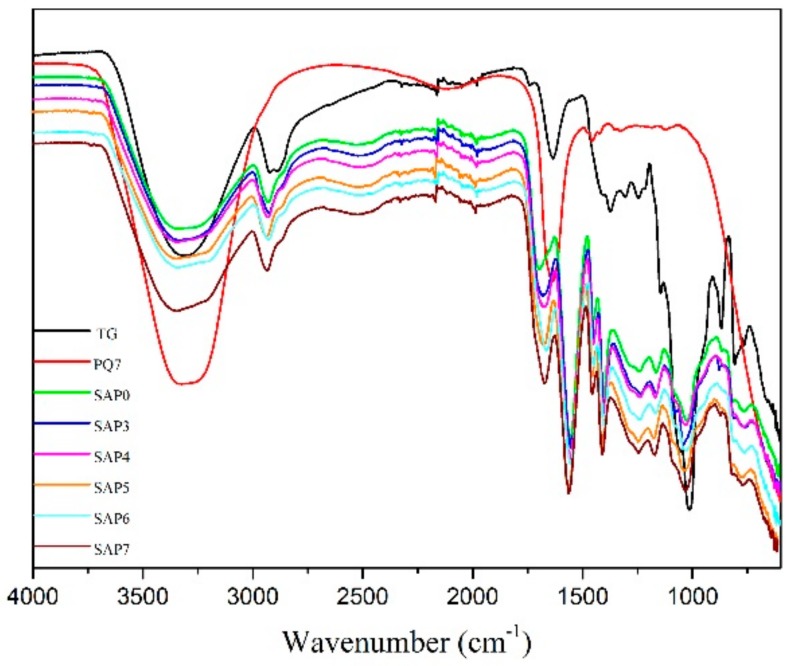
FTIR spectra of TG, PQ7 and all the samples.

The FTIR spectra of all the samples are shown in Figure 3. The bands in the FTIR spectrum of TG appear in two major regions, 3700–2500 cm^−1^ and 1700–700 cm^−1^. The broad band at 3700–3000 cm^−1^ is a result of O-H stretching vibration which is associated with free, inter and intra-molecular bonded hydroxyl groups [35]. The shoulder-shaped band at about 2920 cm^−1^ is the stretching vibration of -CH_2_ [36]. The bands at 1370–1260 cm^−1^ are caused by the bending vibration of the hydroxyl groups. The bands at 1058 cm^−1^ and 1015 cm^−1^ are characteristic of the O-C stretching vibration of the anhydroglucose ring. These bands indicate that TG has the general properties of a polysaccharide. For the spectrum of PQ7, the broad peak at 3500–3000 cm is due to the vibration stretching of N-H. The band at 1650 cm^−1^ is from the amide group in PQ7. The small broad at 1475 cm^−1^ is due to N^+^ bonding with -CH_3_.

For the spectrum of SAP0, the band at 3700–3000 cm^−1^ due to O-H stretching vibration is reduced as some of the hydroxyl groups in TG have been converted to ether linkages by reaction with PAA. The peak at 2930 cm^−1^ is assigned to the stretching vibration of C-H (aliphatic) in the polymer [37]. The band at 1690 cm^−1^ is attributed to the C = O stretching vibration of the carboxyl group in PAA [38]. The peak at 1550 cm^−1^ is assigned to the N-H bending vibration of NMBA in the polymer. A band due to the bending vibration of the -CH_2_ and the asymmetric bending vibration of -CH_3_ appears at 1470–1440 cm^−1^ in SAP0 since the graft polymerization of PAA onto TG has both -CH_2_ and -CH_3_. Compared to the spectrum of TG, the bands observed at about 1370–1260 cm^−1^ are reduced, corresponding to the reduction in hydroxyl groups through graft polymerization. The band at 1250 cm^−1^ is assigned to the stretching vibration of C-O-C due to the graft copolymerization between TG and PAA. The characteristic band of O-C stretching vibration of the anhydroglucose ring at 1058 cm^−1^ and 1015 cm^−1^ is weakened in the presence of the PAA chains. These changes indicate that PAA was grafted on TG.

For the spectrum of SAP3, the band at 3500–3000 cm^−1^ is wider than SAP0 because the N-H stretching vibration in PQ7 overlapped with the O-H stretching vibration. The band at 1690–1650 cm^−1^ becomes wider and stronger because the characteristic absorption of = N^+^ (CH_3_)_2_ at ~1640 cm^−1^ and C = O at ~1660 cm^−1^ in PQ7 [39]. The band at 1470–1440 cm^−1^ is wider because the C-N bonds were introduced after the addition of PQ7. With the PQ7 content increasing from 0.3 g to 0.7 g, the peak intensity at 1690–1650 cm^−1^ and 1470–1440 cm^−1^ gradually increased. These results indicate that PQ7 was not involved in the grafting polymerization PAA on TG molecules.

### 3.4. XRD Analysis

**Figure 4:**
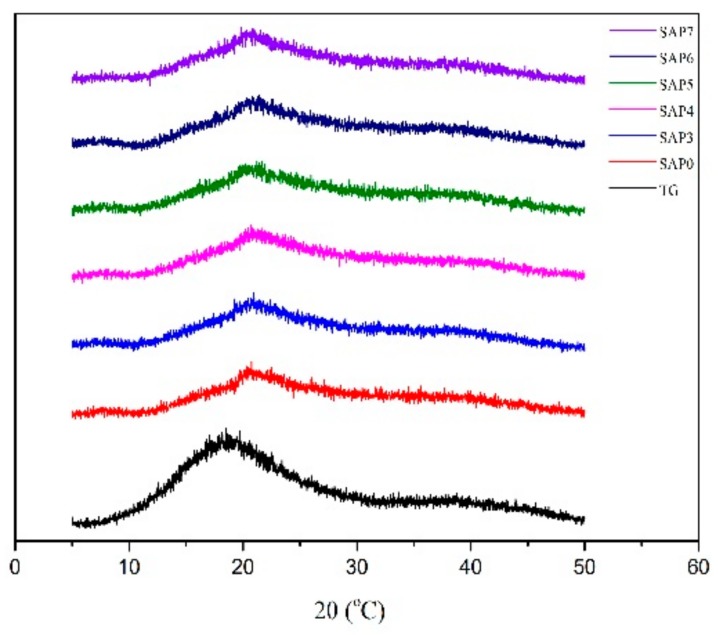
XRD pattern of TG and the SAPs.

Figure 4 displays the XRD patterns of TG and all the SAPs. TG has a peak at 2θ = ~20° which disappeared in the SAPs, indicating that a part of TG molecules were in ordered arrangement, which was interrupted in the grafting process; therefore, the obtained polymer is amorphous. The XRD curves of other SAPs are similar to that of SAP0, which shows that the addition of PQ7 has no effect on the formation of the superabsorbent resin, in which PQ7 interpenetrated in the three-dimensional network, agreeing with the mechanism shown in Figure 1.

### 3.5. Morphological Analysis

**Figure 5:**
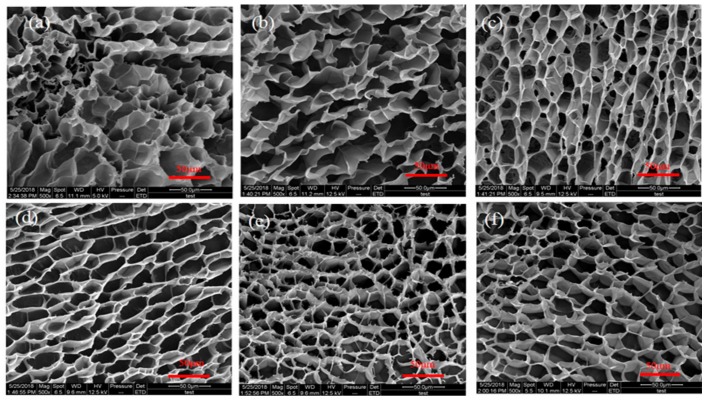
SEM photographs of (**a**) SAP0, (**b**) SAP3, (**c**) SAP4, (**d**) SAP5, (**e**) SAP6, and (**f**) SAP7.

SEM photographs of the SAPs are shown in Figure 5. It can be seen from Figure 5a that the three-dimensional network structure formed via grafting and crosslinking processes of PAA onto TG molecules. As shown in Figure 5b–f, the addition of PQ7 does not affect or destroy the three-dimensional network structure and the result is consistent with the mechanism shown in Figure 1. The three-dimensional network structure is important for water molecules to go into SAP under an osmotic pressure so that the swollen behavior is obvious.

### 3.6. Thermal Stability

**Figure 6:**
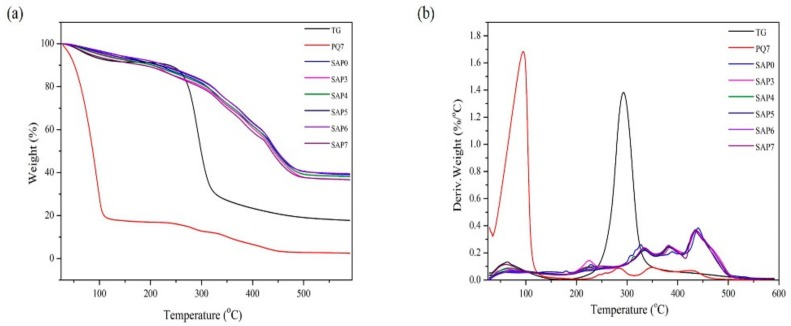
TGA curves (**a**) and DTG curves (**b**) of TG, PQ7 and the SAPs.

Thermogravimetric analysis was used to measure the appropriate temperature range for TG, PQ7 and the SAPs. As shown in Figure 6, TG has two weight loss stages. The first stage occurred in the range of 30–201 °C, where the weight loss was mainly due to water and the weight loss was about 8%. The second stage is in the range of 201–467 °C, where the polysaccharide starts to decompose and the weight loss is 70%. For the curve of PQ7, there is a large weight loss at 25–100 °C, which is due to the fact that the PQ7 used in the experiment is an aqueous solution which contained a lot of water. The second stage (200–380 °C) is due to the decomposition of the quaternary ammonium group, which is the decomposition of the long chain of PQ7 [40].

With SAP0, the first stage occurs at 29–170 °C when the mass loss is due to the presence of moisture and residual organic solvent in the hydrogel with an 11% weight loss which is similar to that of TG and other SAPs. The second period is in the range 170–420 °C where the linear oligomers and other small molecules of the hydrogel begin to be lost. The weight loss at about 420–575 °C is due to the breaking down of the polymer network structure.

For the curves of SAP3 to SAP7, their thermal decomposition trend is similar to that of SAP0, but the final decomposition degree is lower than that of SAP0, which is due to the addition of PQ7. As the content of PQ7 in the hydrogel increases, the weight loss of SAP3 to SAP7 after 200 °C gradually increases, mainly due to the decomposition of PQ7. Based on the above results, it can be seen that the decomposition process of the superabsorbent hydrogel is slow and the decomposition temperature is high, which means that it has good thermal stability.

### 3.7. Antibacterial Test

**Figure 7:**
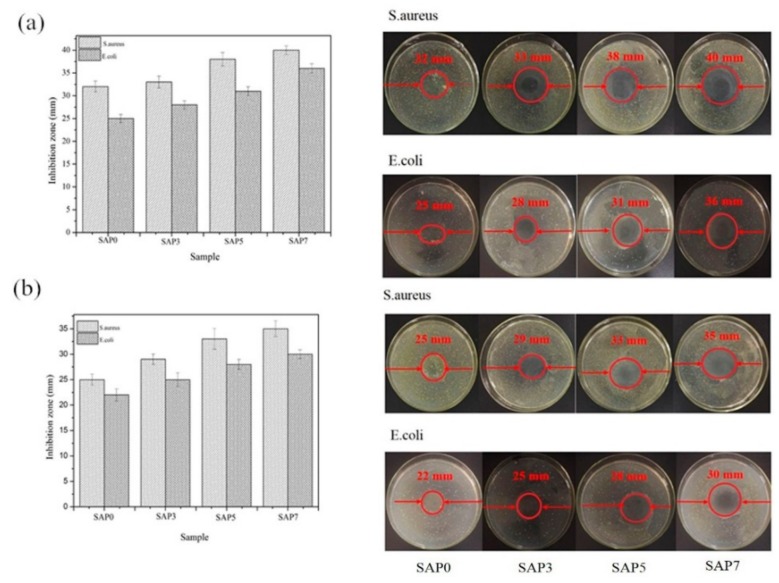
The antibacterial activity of the samples (**a**) and the dried samples after swollen in distilled water (**b**).

PQ7 is a macromolecular long-chain antibacterial substance [41]. Therefore, we studied the antibacterial activity of SAPs containing PQ7. Gram-negative *E. coli* and gram-positive *S. aureus* were selected as model bacteria for the antibacterial activity. The degree of growth inhibition of *S*. *aureus* and *E. coli* by PQ7-free SAP and PQ7-containing SAPs was studied. The results are shown in Figure 7.

As seen in Figure 7a, the inhibition zone size becomes larger with the increasing content of PQ7, indicating that the antibacterial activity of the superabsorbent hydrogel is improved. With an increase in quantity, PQ7 can better inhibit the proliferation and growth of *S. aureus* and *E. coli*, giving the combined hydrogel a good antibacterial effect against *S. aureus* and *E. coli*. For all the SAPs, the ability to inhibit *S. aureus* was better than the ability to inhibit *E. coli*. This is because the lipopolysaccharide layer of *E. coli* is thicker than *S. aureus*, which can protect the bacterial cell wall from being destroyed by PQ7 [42].

The antibacterial activity was tested for the dried hydrogel which had swollen in water, as shown in Figure 7b. The recycle hydrogels have a certain antibacterial effect against *S. aureus* and *E. coli.* It indicates that some PQ7 remains in the hydrogel and exhibits an antibacterial effect in spite of PQ7 being soluble; this is because the long chain structure of PQ7 cannot move freely in the three-dimensional structure of the hydrogel as shown in Figure 1.

### 3.8. Mechnical Property

**Figure 8:**
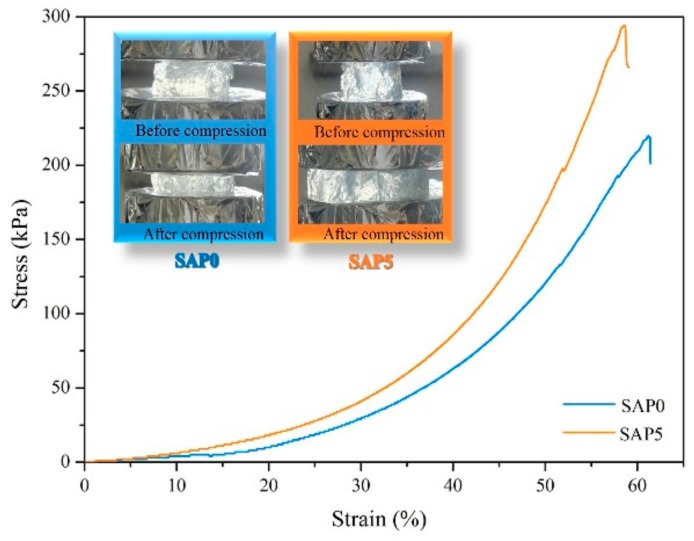
Stress–strain curves of SAP0 and SAP5.

Figure 8 shows compressive stress-strain curves of swollen SAP0 and SAP5 and their photos before and after compression. According to the swelling behavior of SAPs, we chose SAP5 as the test sample. As shown in Figure 8, SAP5 shows a compressive stress of 294 kPa which is higher than that of SAP0 (220 kPa). The addition of PQ7 favors the increase of the hydrogel strength. PQ7 is a polyquaternium with a long molecular chain, which penetrated and interacted with the molecule chains in the three-dimensional networks of the hydrogel. Therefore, the hydrogel was reinforced. The results are in accord with the mechanism shown in Figure 1.

## 4. Conclusions

In this study, an antibacterial hydrogel was synthesized based on TG grafted with PAA and PQ7 chains penetrating the network. The swelling behavior in distilled water and 0.9 wt % NaCl and the antibacterial activity were studied. In addition, the structural details of the superabsorbent hydrogels were determined using FTIR, XRD, SEM and TGA analysis. According to the swelling test, the maximum swelling capacity of the hydrogels in distilled water and 0.9 wt % NaCl solution reached 712 g/g and 68 g/g, respectively. FTIR analysis and XRD studies confirmed the presence of PQ7 in the hydrogel. SEM micrographs revealed that the superabsorbent hydrogel has a honeycomb-like three-dimensional network favoring for water molecules to go into SAP under an osmotic pressure. TGA analysis revealed that it has a good thermal stability, is difficult to decompose, and could be safely used up to 200 °C. The hydrogel had observable antibacterial effects against *S. aureus* and some inhibitory effect against *E. coli* before and after swelling in water. Based on these findings, this antibacterial superabsorbent hydrogel can be a potential candidate for cosmetics and public health.
